# Health of the Army on the Rio Grande

**Published:** 1847-02

**Authors:** 


*Health of the Army on the Rio Grande.*—We learn from Dr. Craig, chief Medical Director, who has been at Monterey ever since Gen. Taylor marched upon that place, that the general health of the army has greatly improved since cool weather set in; though many are still suffering from intermittent fever dysentery and diarrhœa. Dr. Craig says that the surgical operations performed at Monterey, for the most part, did very well. He performed one operation at the shoulder joint, on the field, which recovered. We should be much pleased if the surgeons on duty would keep us regularly advised of the health of the army.—*Ibid.*

